# Efficacy and safety and analysis of thrombopoietin receptor agonists for the treatment of immune thrombocytopenia in adults: analysis of a systematic review and network meta-analysis of randomized controlled trials and results of real-world safety data

**DOI:** 10.3389/fmed.2025.1531824

**Published:** 2025-03-11

**Authors:** Yumeng Liu, Ni Zhang, Tingting Jiang, Yanping Li, Yu Xiong, Yao Liu

**Affiliations:** ^1^Department of Pharmacy, The Daping Hospital of Army Medical University, Chongqing, China; ^2^Department of Pharmacy, Mianyang Central Hospital, University of Electronic, Mianyang, China

**Keywords:** thrombopoietin receptor agonists, immune thrombocytopenia, adults, network meta-analysis, disproportionality analysis

## Abstract

This study aimed to compare the efficacy and safety of thrombopoietin receptor agonists (TPO-RA) in the treatment of immune thrombocytopenia (ITP) in adults. A systematic review was conducted using network meta-analysis and a disproportionality analysis based on the FDA Adverse Event Reporting System (FAERS) database to evaluate hemorrhagic and thrombotic events of clinical concern. Our network meta-analysis included 14 randomized controlled trials (RCTs) involving 1,454 patients. The results indicated that, in terms of efficacy, romiplostim [odds ratio (OR), 0.04; 95% confidence interval (CI), 0 to 0.68] was the most effective, followed by avatrombopag, hetrombopag, and eltrombopag. Regarding safety, there were no significant differences in the safety profiles of the four TPO-RA compared to placebo. According to the surface under the cumulative ranking curve (SUCRA), avatrombopag exhibited the highest safety ranking at 23.8%. Within the FAERS database, we identified 982 cases of TPO-RA-related hemorrhagic and thrombotic events. The highest number of preferred terms (PTs) associated with romiplostim was 26, followed by 18 for eltrombopag and 7 for avatrombopag. The findings of this study suggest that romiplostim exhibits significant efficacy, whereas avatrombopag presents a superior safety profile. In the context of clinical second-line treatment, the selection of the most suitable TPO-RA should be guided by the specific circumstances of each patient.

**Systematic review registration:** https://www.crd.york.ac.uk/PROSPERO/#recordDetails

## Introduction

Immune thrombocytopenia (ITP) is an acquired autoimmune disorder characterized by a low platelet count due to increased platelet destruction and impaired platelet production (1). The prevalence of ITP generally ranges from 2 to 4 cases per 100,000 person-years, with two distinct peaks observed. The first peak occurs between the ages of 20 and 30, with a slight predominance of females, while the second peak is observed after the age of 60, exhibiting an equal sex distribution (2, 3). In adult patients, ITP typically manifests as a chronic acquired disorder. Compared to pediatric patients with ITP, adults are less likely to experience spontaneous remission, have a greater tendency toward hemorrhage, and require more intensive treatment (4).

For adults, the primary objective is to maintain a hemostatic platelet count while minimizing the toxicity of therapy (5). First-line treatment typically involves corticosteroids and intravenous immunoglobulins, whereas second-line treatment generally includes thrombopoietin receptor agonists (TPO-RA), immunosuppressants, rituximab, fostamatinib and splenectomy (6). TPO-RA has been demonstrated to induce megakaryocytic maturation and enhance platelet production, thereby increasing platelet levels and reducing hemorrhage (7). Currently, five types of TPO-RA have been approved for the treatment of adult ITP: avatrombopag, eltrombopag, romiplostim, hetrombopag, and recombinant human thrombopoietin (rhTPO).

Most studies aim to analyze the efficacy and safety of TPO-RA in ITP patients, however, there exists a limited number of investigations that have compared the respective efficacy and safety between different varieties of TPO-RA (8–10). The objective of this study was to analyze the differences in efficacy and safety between various TPO-RA. To this end, a meta-analysis of randomized clinical trials (RCTs) and a disproportionality analysis based on the FDA Adverse Events Reporting System (FAERS) database was conducted.

## Methods

This study was conducted according to the Preferred Reporting Items for Systematic Reviews and Meta-Analysis for Network Meta-Analysis (11). The protocol was registered on PROSPERO (CRD42024506242).

### Search strategy and selection criteria

We searched the PubMed, Embase, Web of Science and Cochrane Central Register of Controlled Trials (CENTRAL) databases on 27 April 2024 using a predetermined strategy. We used the following terms: ‘Purpura, Thrombocytopenic, Idiopathic’or ‘immune thrombocytopenia’ or ‘thrombopoietin receptor agonists’. The explicit search strategies of each database are described in Supplementary material 1. We updated the search literature on 17 July 2024, and screened the reference lists of all included studies, previous meta-analysis and relevant systematic reviews to identify additional studies that were missed in the primary searches.

The literature examining the efficacy and safety of romiplostim, avatrombopag, eltrombopag, and hetrombopag in the treatment of primary immune thrombocytopenia (ITP) was included. The findings encompassed ITP type and duration, platelet counts before and after administration, and the incidence of adverse events (AEs). The inclusion criteria were as follows: (1) Participants diagnosed with primary immune thrombocytopenia for at least 3 months (persistent or chronic ITP); (2) Participants who were adults (aged ≥18). The exclusion criteria included: (1) non-randomized controlled trials; (2) animal studies; (3) studies with incomplete data; and (4) studies for which the original text was unavailable. There were no restrictions on language for the literature.

A disproportionality analysis was conducted by searching the FAERS database for AE reports of avatrombopag, eltrombopag, romiplostim, and hetrombopag from the first quarter (Q1) of 2004 to the third quarter of 2023. The hemorrhage and thrombotic events are defined as specific the preferred term (PT) under the system organ class (SOC) of vascular disorders from the Medical Dictionary for Regulatory Activities (MedDRA) version 25.0. The specific definitions of the hemorrhage events are: haematoma, hemorrhage, internal hemorrhage, haemorrhagic infarction. The specific definitions of the thrombotic events are: thrombophlebitis, embolism venous, peripheral artery thrombosis, thrombophlebitis superficial, thrombosis, pelvic venous thrombosis, jugular vein thrombosis, peripheral embolism, vena cava thrombosis, subclavian vein thrombosis, arterial thrombosis, venous thrombosis, embolism, venous thrombosis limb, deep vein thrombosis, embolism arterial, poor peripheral circulation, aortic thrombosis, blue toe syndrome, and antiphospholipid syndrome.The clinical characteristics of TPO patients with hemorrhage and thrombotic events associated with TPO-RA, including sex, age, reporting year, reporter, and outcome, were collected.

### Data extraction and quality assessment

Two researchers (YL, NZ) independently extracted relevant data from the included studies according to a predefined data extraction form. The following information was extracted: (A) study characteristics, such as the name of the first author, year of publication, study duration, and seven domains of risk of bias assessed according to the Cochrane Handbook 5.1.014 (12); (B) participant characteristics (mean age, proportion of females, duration of illness, platelet count at study entry); (C) intervention details (intervention name, dosing regimen); (D) outcome information, such as duration of response, platelet count after medication, number of AE and number of patients who discontinued medication due to serious adverse events. If the authors directly reported the number of patients responding to the outcome event, it was used directly.

### Date analysis

The primary analysis included descriptive analysis and network meta-analysis for the 2 outcome parameters at postintervention. Data preparation and analysis were performed via Microsoft Excel 2016 for Windows (Microsoft Corporation, Redmond) and R Studio (Version 4.1.1, Boston, MA) using the netmeta package of R.

The ORs were calculated as the effect size of efficacy and safety; both measurements included 95% confidence intervals (CI). We evaluated global heterogeneity and inconsistency in our network model using tau (2) and *I*^2^ statistics and checked for significant within-design heterogeneity and between-design inconsistency via Q statistics (13). In addition, we checked for local consistency in our network using the net splitting method which splits the network estimates into the contributions of direct and indirect evidence (14). We also calculated the SUCRA to rank all interventions. Comparison adjusted funnel plots were generated to detect potential publication bias (15). The level of significance was set, *a priori*, to *p* < 0.05 for all tests.

A disproportionality analysis was conducted utilizing the MySQL database management system, version 8.0.28. The disproportionality was calculated using the reported odds ratios (ROR) and 95% CI. For ROR, it was defined as a significant signal if the lower limit of the 95% confidence interval exceeded 1, with at least 3 cases (16).

## Results

### Study selection

**Figure 1:**
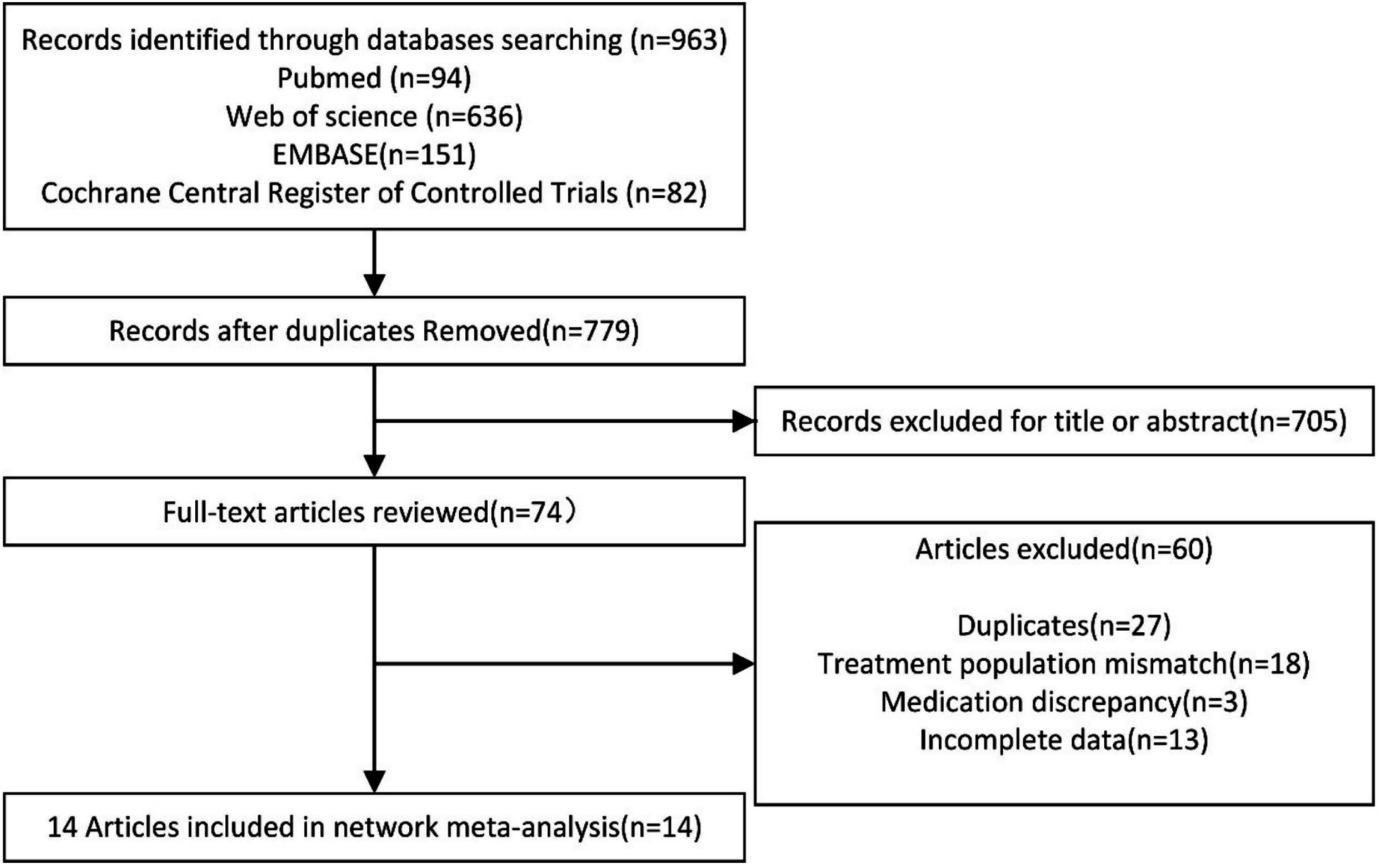
Flowchart of study inclusion.

Following the search strategy, a total of 963 articles were identified. After removing duplicate records using EndNote X9 software, 779 articles remained. The abstracts and titles of all studies were reviewed, resulting in the exclusion of 705 articles that did not meet the specified inclusion criteria. A total of 74 articles were then selected for further investigation. After a thorough examination of the full texts, 14 randomized controlled trials (RCTs) were ultimately included in this network meta-analysis. The selection process is illustrated Figure 1.

### Characteristics of the included trials

**Figure 2:**
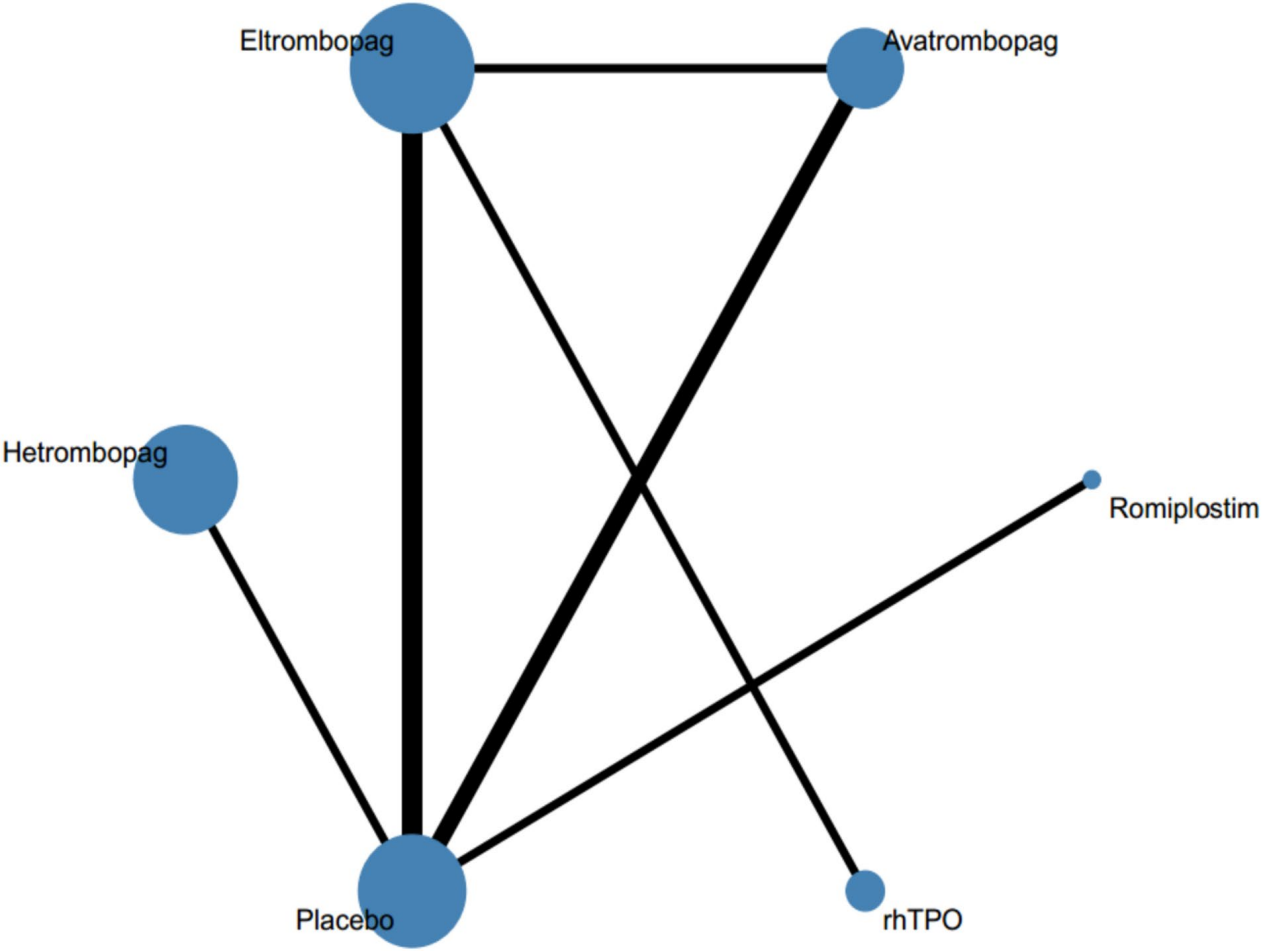
Network of eligible comparisons for all relevant articles. The thickness of the lines between the nodes represents the number of trials comparing every pair of treatments, and the size of the nodes is proportional to the sample number.

The main characteristics of the included studies are summarized in Table 1. All studies were published between 2007 and 2023. A total of 1,454 adults with ITP were included, of whom 1,027 were in the intervention group and 427 in the control group. The duration of the intervention ranged from 2 to 96 weeks and the sample size ranged from 23 to 424 participants. Twelve studies had placebo as the control arm, two other studies where eltrombopag was the intervention group, compared to rhTPO and avatrombopag, respectively. Twelve studies (85.7%) were assessed as low overall risk of bias and two studies (14.3%) had factors with a high risk of bias in the quality assessment of the included articles. Supplementary material 2 was risk of bias assessment for all studies. Figure 2 shows the network map for efficacy.

**Table 1:** Characteristics of included trials.

Author, year	Registration number	Study design	Study duration, weeks	Disease stage	Intervention		Control		Dosage regimen
					Drug	No. of patients	Drug	No. of patients	
Bussel et al. (2007) (2)	NCT00102739	Multicenter, randomized, double-blind, placebo controlled	11	Chronic ITP	Eltrombopag	88	Placebo	29	50 mg orally daily for 6 weeks; dose was adjusted based on platelet counts
Bussel (2009) (3)	NCT00102739	Randomized, double-blind, placebo-controlled, Phase III	6	Chronic ITP	Eltrombopag	76	Placebo	38	50 mg orally daily for 6 weeks; dose was adjusted based on platelet counts
Bussel (2014) (4)	NCT00441090 NCT00625443	Double-blind, randomized, doseranging, placebo-controlled,	24	Persistent and Chronic ITP	Avatrombopag	59	Placebo	5	2.5/5/10/20 mg/day
Cheng (2011) (5)	NCT00370331	Parallel-group, Phase II,	24	Chronic ITP	Eltrombopag	135	Placebo	62	50 mg orally daily for 24 weeks; dose was adjusted based on platelet counts
Huang (2018) (6)	TRA113765	Double-blind, placebo-controlled, Phase III	6	Chronic ITP	Eltrombopag	17	Placebo	18	Initial dose of 25 mg of eltrombopag once day; dose was
Jain (2023) (7)	NCT01438840	Multicenter, randomized, double blind, placebo controlled, Phase III	96	Chronic ITP	Avatrombopag	32	Placebo	17	20 mg initial dose that could be titrated from 5 to 40 mg
Jurczak (2018) (19)	NCT01438840	Multicenter, randomized, double-blind, parallel group phase III	26	Chronic ITP	Avatrombopag	32	Placebo	17	20 mg/day
Mei (2021a) (20)	NCT03771378	Multicenter, Randomized, double blind, placebo controlled, Phase III	2	Chronic ITP	Eltrombopag	48	rhTPO	48	25 mg/day
Mei (2021b) (21)	NCT03222843	Multicenter, randomized, double blind, controlled	24	Chronic ITP	Hetrombopag	339	Placebo	85	2.5/5 mg/day
Mei (2023) (17)	CTR20210431	Multicenter, randomized, double blind, placebo controlled, Phase III	48	Chronic ITP	Avatrombopag	48	Placebo	26	20–40 mg qd
Shirasugi (2011) (22)	NCT00603642	Multicenter, randomized, double-blind, placebo-controlled phase III	12	Chronic ITP	Romiplostim	22	Placebo	12	Starting dose of 3 μg/kg subcutaneously weekly for 12 weeks; dose was adjusted based on platelet counts
Tarantino (2023) (18)	NCT01433978	Double-blind, randomized,Phase III	6	Chronic ITP	Avatrombopag	12	Eltrombopag	11	A:5–40 mg daily E:25–75 mg daily
Tomiyama (2012) (23)	NCT 00540423	Randomized, double-blind, active-controlled, phase III	6	Chronic ITP	Eltrombopag	15	Placebo	8	Starting dose of 12.5 mg (maximum dose of 50 mg) orally daily for 6 weeks; dose was adjusted based on platelet counts
Yang (2017) (24)	NCT 01762761	Randomized, double-blind, placebo-controlled, open-label phase	8	Chronic ITP	Eltrombopag	104	Placebo	51	25 mg once daily for 8 weeks; dose was adjusted based on platelet counts

A total of 982 TPO-RA-related hemorrhage and thrombotic events were identified in the FAERS database between the first quarter of 2004 and the third quarter of 2023. Of these, 541 (55.09%) were associated with romiplostim, 407 (41.45%) with eltrombopag, and 34 (3.46%) with avatrombopag. Hetrombopag, which was included in the network meta-analysis, was not found in the FAERS database. Supplementary material 3 provides a detailed account of the characteristics of patients who experienced TPO-RA-related hemorrhage and thrombotic events.

### Traditional pairwise meta-analysis for efficacy and safety

The results showed that in terms of effectiveness, avatrombopag and eltrombopag were better than placebo, with low heterogeneity (*I*^2^ = 0); in terms of safety, there was no significant difference compared with placebo (*I*^2^ = 19% and *I*^2^ = 6%, respectively). The relevant forest plots are presented in Supplementary material 4.

### Assessment of inconsistency

Fitting the inconsistency model provided no evidence of statistically significant inconsistencies for efficacy and safety (global Wald test: *p* = 0.2999 and *p* = 0.8389, respectively). And there was no evidence of local inconsistency (*p* = 0.2785 and *p* = 0.8219, respectively). The relevant forest plots are presented in Supplementary material 5.

### Results of the main outcome measures

#### Effectiveness results

In terms of efficacy, romiplostim (OR, 0.04; 95% CI, 0 to 0.68), avatrombopag (OR, 0.15; 95% CI, 0.08 to 0.28), hetrombopag (OR, 0.19; 95% CI, 0.11 to 0.34), and eltrombopag (OR, 0.19; 95% CI, 0.19 to 0.35) were more effective than placebo (Table 2). The analysis outcome of SUCRA showed no significant differences between the drugs. The most effective treatment is romiplostim (92.9%), followed by avatrombopag (82.5%), hetrombopag (52.8%) and eltrombopag (51.2%) (Table 3).

**Table 2:** Network meta-analysis of efficacy.

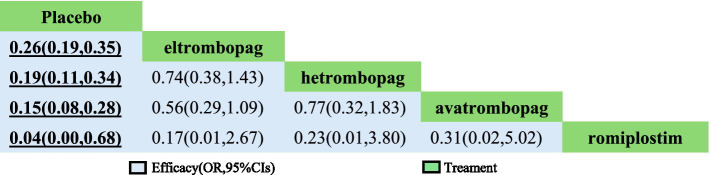

**Table 3:** Treatment ranking for efficacy.

Treatment	SUCRA
Romiplostim	92.9
Avatrombopag	82.5
Hetrombopag	52.8
Eltrombopag	51.2
Placebo	0.3

#### Safety results

There was no significant difference between the four drugs and the placebo control in terms of safety (Supplementary material 6). SUCRA ranking results showed that avatrombopag was the best safety profile (Table 4).

**Table 4:** Treatment ranking for safety.

Treatment	SUCRA
hetrombopag	59.5
romiplostim	53.1
Placebo	42.6
eltrombopag	42.5
avatrombopag	23.8

The disproportionality results of ROR were shown in Table 5. The greatest number of PTs identified for romiplostim was 26, followed by 18 for eltrombopag and finally 7 for avatrombopag. The most frequently reported PT for romiplostim and avatrombopag was deep vein thrombosis, with 185 and 75 cases, respectively. The largest number of cases reported for eltrombopag was for hemorrhage (147).

**Table 5:** Disproportionality analysis in FAERS database.

PT	Romiplostim		Eltrombopag		Avatrombopag	
	a	ROR (95% CI)	a	ROR (95% CI)	a	ROR (95% CI)
Hemorrhage events						
Haematoma	39	3.49 (2.55, 4.78)	36	3.46 (2.49,4.79)	/	
Hemorrhage	161	4.87 (4.17, 5.69)	147	4.76 (4.05,5.60)	5	2.58 (1.07,6.21)
Internal hemorrhage	/		14	3.06 (1.81,5.16)	/	
Haemorrhagic infarction	/		3	14.12 (4.53,44.02)	/	
Thrombotic events						
Thrombophlebitis	4	2.80 (1.05, 7.47)	6	4.51 (2.02,10.05)	/	
Embolism venous	3	3.32 (1.07, 10.31)	/	/		
Peripheral artery thrombosis	3	4.60 (1.48, 14.29)	/	/		
Thrombophlebitis superficial	6	3.66 (1.64, 8.15)	5	3.25 (1.35,7.83)	/	
Thrombosis	67	2.47 (1.95, 3.15)	97	3.85 (3.15,4.70)	7	4.17 (1.98,8.77)
Pelvic venous thrombosis	4	5.57 (2.08, 14.87)	/	/		
Jugular vein thrombosis	5	6.63 (2.75, 15.96)	/	/		
Peripheral embolism	4	8.17 (3.06, 21.83)	/	/		
Vena cava thrombosis	5	7.73 (3.21, 18.63)	/	/		
Subclavian vein thrombosis	5	10.57 (4.38, 25.48)	/	/		
Arterial thrombosis	7	9.28 (4.41, 19.52)	6	8.54 (3.82,19.05)	/	
Venous thrombosis	12	8.06 (4.57, 14.22)	4	2.86 (1.07,7.64)	/	
Embolism	23	8.90 (5.91, 13.42)	14	5.80 (3.43,9.80)	3	19.12 (6.15,59.43)
Venous thrombosis limb	11	11.13 (6.15, 20.16)	6	6.48 (2.90,14.45)	/	
Deep vein thrombosis	185	7.15 (6.18, 8.27)	75	3.08 (2.46,3.87)	9	8.38 (4.35,16.15)
Embolism arterial	13	23.50 (13.58, 40.67)	4	7.61 (2.85,20.33)	/	
Poor peripheral circulation	/		4	2.92 (1.09,7.78)	/	
Aortic thrombosis	/		4	9.29 (3.47,24.83)	/	
Blue toe syndrome	/		3	21.53 (6.88,67.34)	/	
Antiphospholipid syndrome	/		/		3	79.81 (25.60,248.80)

#### Publication bias and sensitivity analysis

Comparison-adjusted funnel plots were used to assess publication bias. The comparison-adjusted funnel plots for efficacy and safety were relatively symmetrical, and no obvious publication bias was found (Supplementary material 7).

#### Meta-regression and subgroupanalysis

Meta-regression and subgroupanalysis were performed by publication year, age, female percentage, trial duration, whether to perform splenectomy. The results showed that all interacting terms were non-significant (Table 6).

**Table 6:** Meta-regression and subgroupanalysis.

Moderators	Interaction term (B)	95% lower limit	95% upper limit	Heterogeneity (σ)
Publication year	−0.18	−0.42	0.02	0.92
Age	−0.16	−0.36	0.07	1.04
Trial duration	0.02	−0.18	0.23	1.37
Female percentage	0.09	−0.72	0.94	1.26
Whether to perform splenectomy (1 = YES; 0 = NO)	0.54	−2.01	3.04	1.18

## Discussion

This study employed a network meta-analysis and disproportionality analysis based on the FAERS database to compare the efficacy and safety of four TPO-RA: romiplostim, avatrombopag, eltrombopag, and hetrombopag, as recommended by current guidelines for the treatment of ITP in adults. This network meta-analysis incorporates the most recent studies compared to previous meta-analyses and includes a larger patient population, providing higher statistical power than traditional pairwise meta-analyses. Additionally, it incorporates AEs data from real-world patients to further assess the safety of TPO-RA (2–7, 17–24).

This network meta-analysis demonstrated the overall efficacy and safety of romiplostim, avatrombopag, eltrombopag, and hetrombopag in the treatment of ITP, which is largely consistent with the findings of previous meta-analyses. ITP is an acquired autoimmune disease characterized by isolated thrombocytopenia, resulting from increased platelet destruction and decreased platelet production, both of which are mediated by immune disorders (25). The primary mechanism underlying ITP is the loss of immune tolerance to platelet autoantigens, leading to aberrant activation of both humoral and cellular immunity. These factors collectively contribute to accelerated platelet destruction and insufficient production of platelets by megakaryocytes (26). TPO-RA primarily increase platelet counts by binding to their receptor, the megakaryocyte progenitor cell receptor (MPL). MPL is a receptor tyrosine kinase that is highly expressed on the surface of megakaryocytes and platelet precursor cells. By binding to MPL, TPO-RA stimulates megakaryocyte proliferation and differentiation, thereby promoting platelet maturation and release (27). The results of this study differ from those of the network meta-analysis conducted by Liu (1), in which avatrombopag (87.5%) was found to be more effective than romiplostim (71.1%). This discrepancy in findings may be attributed to the inclusion of three recently published RCTs on avatrombopag in 2023 (7, 17, 18), which resulted in a shift in the ranking of avatrombopag and romiplostim in terms of efficacy. It has been demonstrated that neither avatrombopag nor romiplostim interacts with food or drugs. Eltrombopag represents the inaugural oral TPO-RA for the treatment of ITP. hetrombopag is a highly selective TPO-RA that has been developed through a series of modifications based on the structure of eltrombopag. This new agent exhibits greater activity and a reduced incidence of AE (28). However, they are susceptible to interactions with calcium-rich foods and antacids containing aluminum and magnesium, which can significantly reduce the concentration of the active ingredient and, consequently, its efficacy (29).

Regarding safety, our systematic review indicated that there was no significant difference in the incidence rate of AEs among patients in the four drug and placebo groups. This study is in accordance with the results of the traditional meta-analysis conducted by Shen (30). Even if there is no statistically significant difference in safety, their use still needs to be cautious. Regarding the most common thrombotic AE associated with ITP, no significant difference was observed compared to placebo. The SUCRA analysis indicates that among the four drugs, avatrombopag (23.8%) has the best safety profile, followed by eltrombopag (42.5%), romiplostim (53.1%), and hetrombopag (59.5%). The FAERS database was utilized to analyze the occurrence of AEs, particularly focusing on hemorrhagic and thrombotic events of greatest clinical concern. Among the four drugs, romiplostim reported the highest number of adverse events (55.09%), while avatrombopag reported the lowest (3.46%). However, it would be misleading to conclude that romiplostim has a high incidence of AEs and poor safety. This perception may be attributed to its earlier market entry, which occurred 10 years prior to the launch of avatrombopag in 2018 (31, 32). A review of AE reports indicates that avatrombopag has a lower incidence of hemorrhagic and thrombotic events since its launch, with a relatively limited number of AEs occurring concurrently. The most frequently reported AE associated with both romiplostim and avatrombopag is deep vein thrombosis. The occurrence of deep vein thrombosis is related to several factors, including a history of venous thromboembolism, coronary artery disease, atrial fibrillation, and other conditions. Nevertheless, the guidelines do not prohibit the use of TPO-RA in this population, and therefore, the use of TPO-RA is not considered to increase the risk of deep vein thrombosis in patients. The most frequently reported AE associated with eltrombopag is hemorrhage, which is often a result of the drug’s ineffectiveness. As a medication for the treatment of thrombocytopenia, eltrombopag increases platelet counts by stimulating platelet production in the bone marrow, with the majority of the drug being metabolized by the liver (33). The initial dosage is calculated according to the patient’s weight. The platelet count is monitored on an individual basis in order to ascertain the optimal maintenance dose. The effects of treatment may take several weeks or even months to manifest (34). Concurrently, concomitant administration of proton pump inhibitors (35), H2 receptor antagonists (36), anticoagulants (37) and liver enzyme inducers will result in a reduction in the efficacy of the drug. A review of the reported hemorrhage events associated with eltrombopag suggests that the drug’s efficacy may be compromised by factors such as dosage, the use of concomitant medications, and other variables that fall outside the standard treatment parameters.

The results of this study indicate that among TPO-RA, romiplostim exhibits superior efficacy, followed by avatrombopag. Additionally, avatrombopag demonstrates a favorable safety profile within the TPO-RA category. Findings from the FAERS database analysis further support the conclusion that avatrombopag offers a favorable safety profile among TPO-RA. As an oral formulation, avatrombopag is more convenient to use than romiplostim, which requires injection. Furthermore, it is less likely to interact with food and other medications. Based on these findings, avatrombopag presents distinct advantages in the second-line treatment of ITP in adults, providing new evidence for the clinical use of TPO-RA in this context.

This study has several limitations. First, the number of studies included in the systematic review is limited, and various types of interventions are employed, which restricts the interpretation of the research results. Second, each trial utilizes different definitions of response criteria. Additionally, the dose and duration of treatment were not standardized in this study. Most of the included RCTs adjusted drug dosages based on individual patient conditions, which may lead to variability in platelet outcomes. Furthermore, discrepancies exist in the definitions of AEs, potentially introducing heterogeneity into the analysis. Finally, the FAERS database, as a global spontaneous AE reporting system, has inherent biases. Moreover, due to the lack of information on healthy patients, the incidence of drug-related AEs cannot be accurately calculated. Hetrombopag, as a newly launched drug in China, is not recorded in the FAERS database and therefore lacks this data.

## Conclusion

The data derived from the systematic review and network meta-analysis suggest that among TPO-RA, romiplostim exhibits robust efficacy, whereas avatrombopag distinguishes itself with favorable safety profiles. When integrating evidence from both the network meta-analysis and the FAERS database, avatrombopag emerges with specific advantages as a second-line treatment option for ITP in adults among TPO-RA. Nevertheless, further controlled head-to-head RCTs are imperative to augment the evidence base for the clinical management of ITP.

## Data Availability

The raw data supporting the conclusions of this article will be made available by the authors, without undue reservation.
