# Supplementary material for: Efficacy and safety and analysis of thrombopoietin receptor agonists for the treatment of immune thrombocytopenia in adults: analysis of a systematic review and network meta-analysis of randomized controlled trials and results of real-world safety data
Source: Front Med (Lausanne). 2025 Mar 11;12:1531824. doi: 10.3389/fmed.2025.1531824 (PMC11974604; doi:10.3389/fmed.2025.1531824)
Supplement: Supplementary file 1 [file Data_Sheet_1.docx]

***Supplemental 1.search strategies***

**PUBMED**

("Purpura, Thrombocytopenic, Idiopathic" [Mesh]) AND ((((((thrombopoietin receptor agonists [Title/Abstract]) OR Romiplostim [Title/Abstract] )OR Avatrombopag [Title/Abstract] )OR Eltrombopag [Title/Abstract])OR Hetrombopag[Title/Abstract] ）OR Thrombopoietin Receptor Agonists[Title/Abstract]) AND (((((Randomised Controlled Trials[Title/Abstract]) OR Clinical Controlled Trials[Title/Abstract] OR Clinical Controlled Trials[Title/Abstract]) OR Randomised[Title/Abstract]) OR Controlled[Title/Abstract]) OR Randomised[Title/Abstract])

**EMBASE**

#1 'thrombopoietin receptor agonists':ti,ab,kw

#2 (romiplostim*:ti,ab OR avatrombopag*:ti,ab OR eltrombopag:ti,ab OR hetrombopag*:ti,ab OR thrombopoietin) AND receptor AND agonists*:ti,ab

#3 #1 AND #2

#4 (random* OR controlled):ti,ab

#5 #3 AND #4

**Web of science**

TS=(immune thrombocytopenia OR Purpura, Thrombocytopenic, Idiopathic) AND TS=( romiplostim* OR avatrombopag* OR eltrombopag OR hetrombopag* OR thrombopoietin receptor agonists*) AND TS=(random* OR controlled)

**Cochrane Central Register of Controlled Trials**

#1 MeSH descriptor: [Purpura, Thrombocytopenic, ldiopathic] this term only

#2 (romiplostim* OR avatrombopag* OR eltrombopag OR hetrombopag* OR thrombopoietin receptor agonists*).ti, ab,kw in Trials

#3 #1 AND #2

#4 (random* OR controlled):ti,ab,kw in Trials

#5 #3 AND #4

*
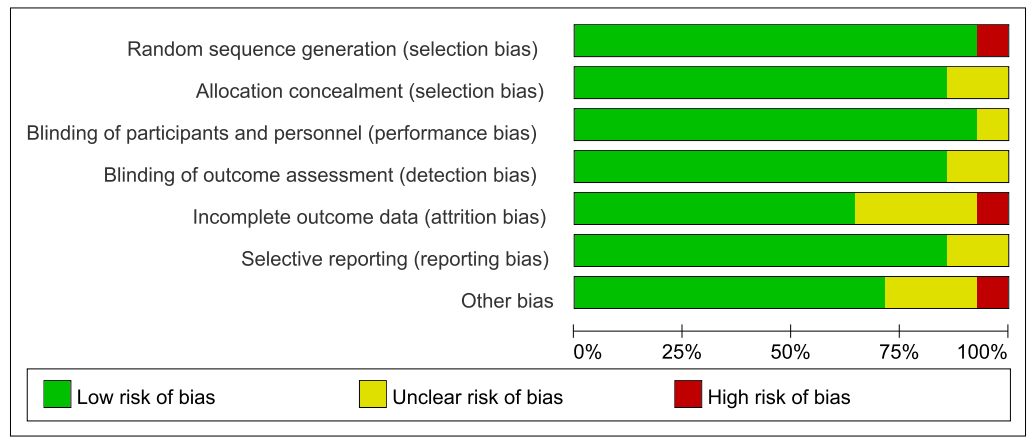
****Supplemental 2. Risk of bias assessmen tfor all studies***

**Figure 1.** **Risk of bias graph**

***
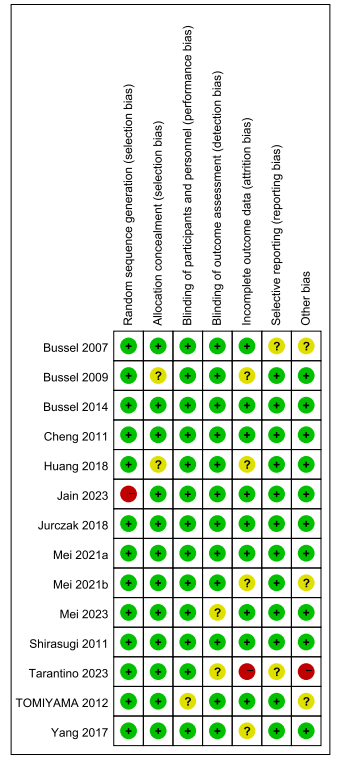
***

**Figure 2.** **Risk of bias summary**

***Supplemental 3.* characteristics of patients**

**Table1 The characteristics of patients with TPO-related haemorrhage and thrombotic events**

| **Characteristics** | Romiplostim | Eltrombopag | Avatrombopag |
| --- | --- | --- | --- |
| **case number** | 522 | 403 | 30 |
| **Gender** |  |  |  |
| Female | 289 | 245 | 16 |
| Male | 226 | 152 | 14 |
| unkown | 7 | 6 | 0 |
| **Age(years)** |  |  |  |
| 18-44 | 90 | 76 | 6 |
| 45-64 | 191 | 147 | 7 |
| 65-74 | 100 | 77 | 7 |
| ≥75 | 141 | 103 | 10 |
| **Reporting year** |  |  |  |
| 2008 | 0 | 0 | 0 |
| 2009 | 0 | 7 | 0 |
| 2010 | 57 | 32 | 0 |
| 2011 | 127 | 43 | 0 |
| 2012 | 49 | 26 | 0 |
| 2013 | 34 | 7 | 0 |
| 2014 | 47 | 20 | 0 |
| 2015 | 42 | 25 | 0 |
| 2016 | 28 | 12 | 0 |
| 2017 | 25 | 14 | 0 |
| 2018 | 22 | 14 | 0 |
| 2019 | 28 | 6 | 1 |
| 2020 | 12 | 19 | 4 |
| 2021 | 28 | 34 | 1 |
| 2022 | 15 | 76 | 4 |
| 2023 | 8 | 68 | 20 |
| **Reporter** |  |  |  |
| Other health-professional | 135 | 73 | 8 |
| Consumer | 62 | 197 | 11 |
| Physician | 307 | 110 | 11 |
| Pharmacist | 18 | 23 | 0 |
| **Outcome** |  |  |  |
| Death | 30 | 33 | 0 |
| Life-threatening | 8 | 15 | 3 |
| Hospitalization | 141 | 99 | 11 |
| Disability | 5 | 4 | 0 |
| other | 338 | 252 | 16 |

**
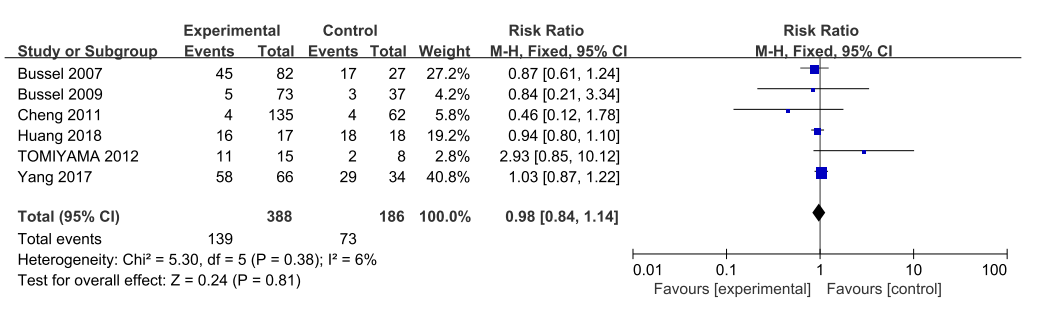
*Supplemental 4. Traditional pairwise meta-analyses for efficacy and safety***

**Figure 1.** **Forest plot comparing the efficacy of eltrombopag and placebo**

**
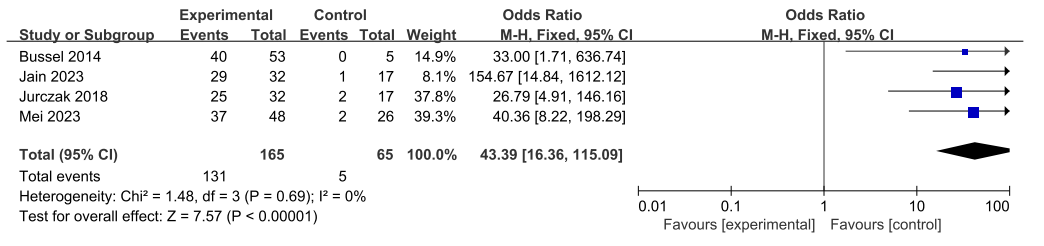

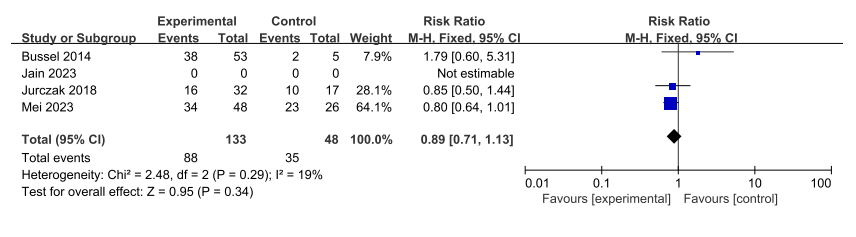
Figure 2. Forest plot comparing the efficacy of Avatrombopag and placebo**

***
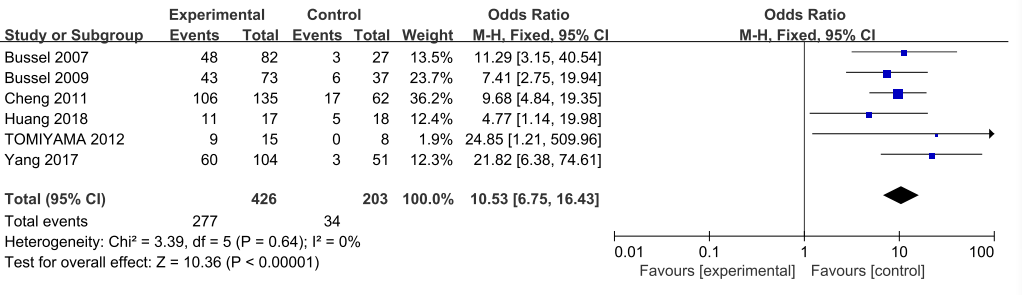
*Figure 3. Forest plot comparing the safety of eltrombopag and placebo**

**Figure 4. Forest plot comparing the safety of Avatrombopag and placebo**

**
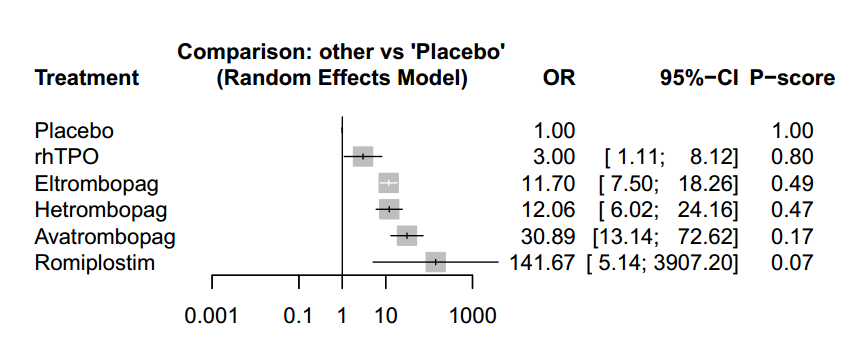
*Supplemental 5. Assessment of inconsistency***

**Figure 1. Forest plot of global inconsistency assessment of efficacy**

**
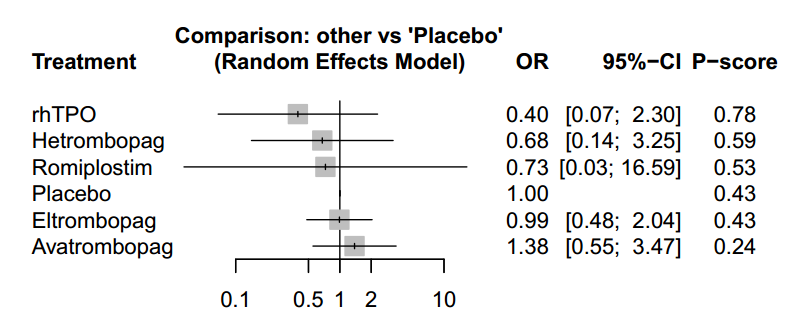
Figure 2. Forest plot of global inconsistency assessment of safety**

**
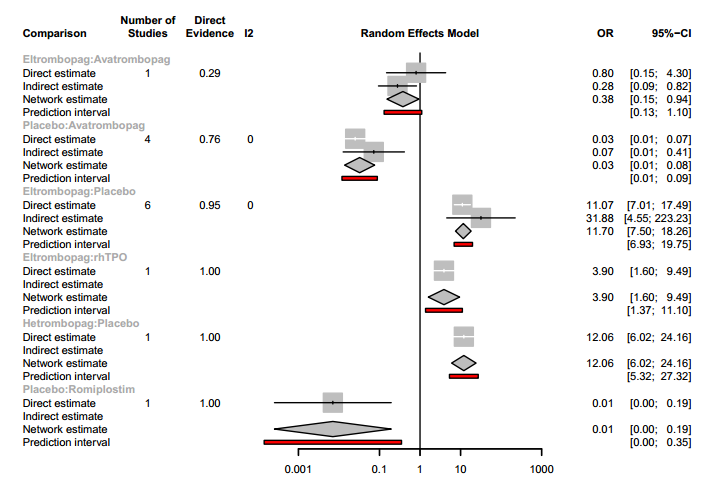
**

**Figure 3. Forest plot of local inconsistency assessment of efficacy**

**
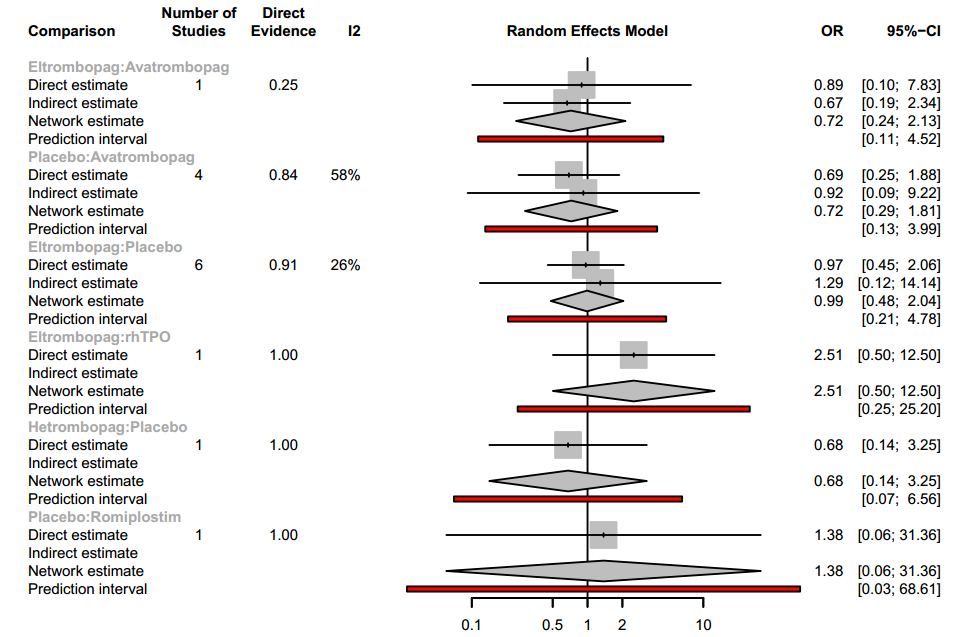
**

**Figure 4. Forest plot of local inconsistency assessment of safety**

***Supplemental 6.* Network meta-analysis of safety**

| **Avatrombopag** |  |  |  |  |
| --- | --- | --- | --- | --- |
| 0.72(0.24,2.13) | **Eltrombopag** |  |  |  |
| 0.72(0.29,1.81) | 1.01(0.49,2.08) | **Placebo** |  |  |
| 0.53(0.02,13.68) | 0.73(0.03,18.17) | 0.73(0.03,16.59) | **Romiplostim** |  |
| 0.49(0.08,3.01) | 0.68(0.12,3.84) | 0.68(0.14,3.25) | 0.93(0.03,30.81) | **Hetrombopag** |
|  | Efficacy(OR,95%CIs) |  | Treament |  |

***Supplemental 7. Figure that shows the comparison-adjusted funnel plot for each outcome***

**
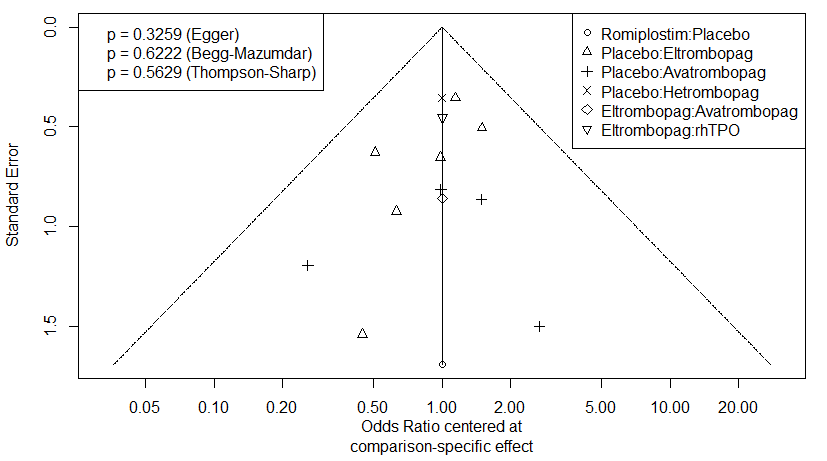
**

**
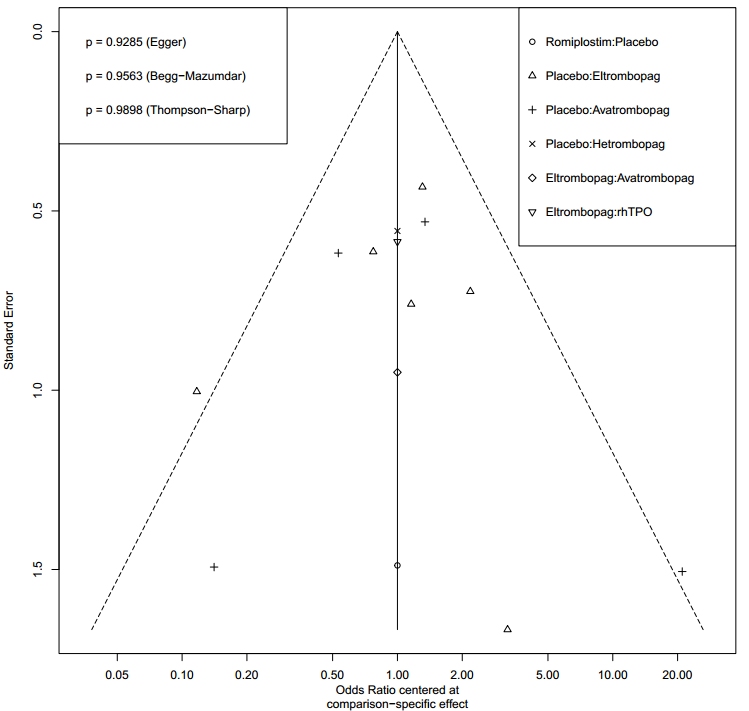
Figure 1.comparison-adjust funnel plot for efficacy**

**Figure 2.comparison-adjust funnel plot for safety**
